# Selecting lines for spectroscopic (re)measurements to improve the accuracy of absolute energies of rovibronic quantum states

**DOI:** 10.1186/s13321-021-00534-y

**Published:** 2021-09-16

**Authors:** Péter Árendás, Tibor Furtenbacher, Attila G. Császár

**Affiliations:** 1 Budapest Business School, Budapest, Hungary; 2 Institute of Chemistry, ELTE Eötvös Loránd University, Budapest, Hungary; 3 ELKH-ELTE Complex Chemical Systems Research Group, Budapest, Hungary

**Keywords:** High-resolution molecular spectroscopy, Spectroscopic networks, Graph theory, Accurate rovibronic energies

## Abstract

Improving the accuracy of absolute energies associated with rovibronic quantum states of molecules requires accurate high-resolution spectroscopy measurements. Such experiments yield transition wavenumbers from which the energies can be deduced *via* inversion procedures. To address the problem that not all transitions contribute equally to the goal of improving the accuracy of the energies, the method of Connecting Spectroscopic Components (CSC) is introduced. Using spectroscopic networks and tools of graph theory, CSC helps to find the most useful target transitions and target wavenumber regions for (re)measurement. The sets of transitions suggested by CSC should be investigated by experimental research groups in order to select those target lines which they can actually measure based on the apparatus available to them. The worked-out examples, utilizing extensive experimental spectroscopic data on the molecules H$_2^{~16}$O, $^{32}$S$^{16}$O$_2$, H$_2^{~12}$C$^{16}$O, and $^{14}$NH$_{3}$, clearly prove the overall usefulness of the CSC method and provide suggestions how CSC can be used for various tasks and under different practical circumstances.

**Supplementary Information:**

The online version contains supplementary material available at 10.1186/s13321-021-00534-y.

## Introduction

Due to their constant development, experimental high-resolution molecular spectroscopic techniques [1] yield an ever-increasing amount of more and more precise and accurate information about the dynamics of molecules. One of the principal driving forces behind many of the spectroscopic advances and the improved measurements is the dream of the complete characterization of the rovibronic spectra of molecules in various environments and under assorted conditions.

Measurements and first-principles calculations lead directly to wavenumbers, intensities, and lineshapes. In this paper we are addressing only part of the information provided by the extremely complex measured spectra, namely the position of the rotational-vibrational-electronic (rovibronic) lines, arising from transitions among the quantum states of the molecule.

Understanding the measured spectra and the underlying dynamics necessitates the determination of the non-measurable energy-level structure of the quantum states. However, even simple triatomic molecules have bound rovibrational quantum states on the order of millions and the number of possible transitions is on the order of billions. Moreover, to obtain information about the energy-level structure of a molecule in a field-free environment, we have various experimental setups corresponding to different measurable transitions. Making advances related to the understanding of the energy-level structure and the transitions and making sure that the knowledge gained is as accurate and precise as possible and feasible, requires sophisticated methods. It seems to the authors that quantum theory alone is not able to provide a full solution to these problems, and that in the fourth age of quantum chemistry [2] it is graph (network) theory that can help experimental as well as theoretical spectroscopists to make further significant advances.

**Fig. 1:**
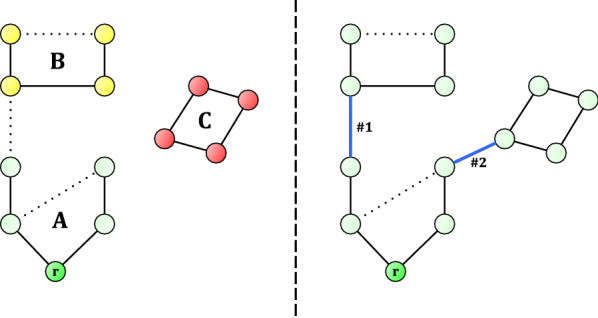
*Left-hand graph*: a small spectroscopic network, with vertex *r* representing the root. Solid black edges represent accurate transitions, dotted ones represent transitions with insufficient accuracy. Observe that quantum states in subgraph **A** (green vertices) have accurate energies, energies in subgraph *B* (yellow vertices) depend on the inaccurate edge between **A**, **B**, while absolute energies in the detached subgraph **C** (red vertices) are unavailable. *Right-hand graph*: by adding two accurate edges, those in blue, all the energies of the vertices would be known with high accuracy

Let us recall briefly the connection between rovibronic energies and transition wavenumbers. If the energy of quantum state *A* is *E*(*A*), and there is a transition from quantum state *A* to quantum state *B* with a wavenumber of ${\tilde{\nu }}(AB)$, then the energy of *B* is $E(B)=E(A)+{\tilde{\nu }}(AB)$ (among spectroscopists, it is widely accepted to refer to this statement as the Ritz principle [3]). Taking advantage of the fact that the energy of the lowest-energy state of the molecule can be defined, without loss of generality, to be zero, the transition wavenumbers measured determine the absolute energies of at least some of the quantum states of at least one of the nuclear-spin isomers of the given molecule.

Transition wavenumbers provided by either measurements or theoretical calculations have a corresponding uncertainty: a wavenumber *w* with an uncertainty *u* means that the ‘real’ wavenumber of the transition lies in the $(w-u,w+u)$ interval with a probability of $95\%$. Consequently, the energy values determined using the extended Ritz principle are also subject to the wavenumber uncertainties in the database.

It has been usual practice to collect measured transition data, including wavenumbers, intensities, and lineshapes, into line-by-line (LBL) databases, such as the HITRAN [4] and the ReSpecTh [5] spectroscopic information systems. It is also usual practice, partially based on the need and the anticipation of users of LBL databases, that spectroscopic data sets collated from the literature may contain not only transitions of experimental origin but also transitions that come from first-principles (quantum) calculations or modeling efforts utilizing effective Hamiltonians.

Let us add at this point an important note about the utilization of spectroscopic data, for example of the data stored in the aforementioned spectroscopic databases. From the point of view of applications, like atmospheric modeling [6, 7], remote sensing and retrievals [8–10], determination of temperature-dependent partition functions [11–13], and derivation of equations of state [14], both the transition wavenumbers and the underlying energies are required.

Now we are ready to state the pivotal premise that forms the basis of this paper: each new transition in a database serves equally the goal of expanding accurate wavenumber data, but not all transitions contribute equally to the goal of expanding accurate energy data. To elaborate this point, let us take a look at the left-hand graph of Fig. 1, which represents a tiny spectroscopic database *via* its spectroscopic network [15, 16]. This database contains 14 transitions which span 13 quantum states. The solid edges of Fig. 1 are considered to be accurately known, while the dotted edges represent ‘inaccurate’ transitions: inaccurate transitions have an uncertainty larger than a threshold value chosen. Observe that the energy of the green quantum states (subgraph *A*) can be determined by using only accurate transitions. The accuracy of the yellow states (subgraph *B*) depend on the inaccurate transition connecting subgraphs *A* and *B*. As there is no path from the root to the red states, the absolute energies in subgraph *C*, also called a floating component [17], can only be determined after fixing the energy of one of the red states first (which will then act as a pseudo-root), for example, using first-principles computations. This also means that all red-state energies inherit the uncertainty of the first-principles computations.

**Fig. 2:**
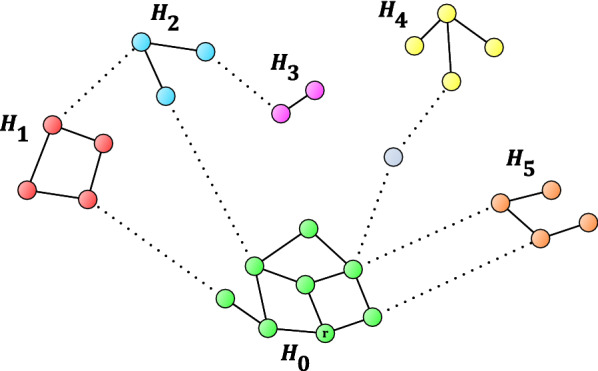
An example graph *G*, showing various scenarios how to reach the other subgraphs from the root through external edges. Solid edges: internal transitions. Dotted edges: external edges. Vertex *r* is the root of graph G

Note that depending on the method used to determine the energy values, the energy of the green quantum states of Fig. 1 might actually receive a larger (*i.e.*, worse) uncertainty than the uncertainty of the edges in subgraph *A*. Therefore, having a path of accurate edges from the root to the selected state is assumed to act as a *necessary* condition to obtain an accurate energy level of the same magnitude of the edges; in other words, it is not assumed to be a *sufficient* condition. Real database examples (see the *Practical examples* section) show that this necessary condition does not hold for large sets of quantum states in various LBL datasets. This paper is about an efficient method that extends the *necessary* condition to these large sets of quantum states.

Both sources of inaccurate absolute energy values shown in Fig. 1 are, of course, well known in the literature [17, 18]. The right-hand graph of Fig. 1 shows a possible solution for both problems. By remeasuring the transition that connects subgraphs *A* and *B* (transition #1), it becomes viable to determine the absolute energies of the previously yellow states using only accurate transitions. By conducting a new measurement, yielding the transition connecting subgraph *A* to subgraph *C* (transition #2), the energies of the previously red states may also be determined accurately. These two new edges are shown in blue in Fig. 1.

In a practical scenario, finding a suitable set of new transitions to connect the subgraphs is not an easy task, especially when tens of thousands of transitions form the original graph and there are also tens of thousands of new, potential transitions. The main result of this paper is the method of Connecting Spectroscopic Components (CSC), which provides a ranking of transition sets based on their usefulness when added to the original database.

Since this paper heavily relies on graph theory, which might be an unfamiliar field for some of the readers, the authors would like to recommend two excellent textbooks for reference: one by Lovász, Vesztergombi, and Pelikán [19] and another one by Newman [20].

## The method of connecting spectroscopic components (CSC)

### Input

We need two sets of transitions characterizing the same molecule as input to the CSC method. Let us refer to the first set as *internal* transitions (*i.e.*, they are *in* the database we would like to improve), and to the second set as *external* transitions (*i.e.*, these come from external sources, for example, from new measurements). The idea is to add transitions from the external transition set to the database of internal transitions. However, expanding the database with external transitions has a cost. Thus, it is desirable to classify the external transitions based on their usefulness when added to the set of internal transitions.

Let us investigate two characteristic examples of transition sets.

#### Example 1

The *internal transitions* are chosen from the complete spectroscopic database of a molecule, for example, entries in the ReSpecTh [5] or HITRAN [4] database. The corresponding *external transitions* are transitions that could be measured in a new experiment. The external transitions could be identified by investigating the output of first-principles computations.

#### Example 2

The *internal transitions* are transitions of a spectroscopic database that are under a chosen upper uncertainty threshold, for example, $10^{-3}$ cm$^{-1}$. The *external transitions* are the ones that could be added to the database and that are under the uncertainty threshold (provided, for example, by the precision of the new measurement or the theoretical calculations).

### Graph construction

First, let us build the spectroscopic network using only the set of internal transitions. Let us call this graph *H*. Let us denote, throughout this study, the vertex representing the root of *H* by *r*. (In practice, it is safe to assume that there is at least one internal transition connected to the root. Else, this method is consistent by adding the root as an isolated vertex.) The graph *H* might have multiple connected components. Let us denote the connected component that contains *r* by $H_0$.

If *H* has only one connected component, $H_0$, then each external transition would contribute by adding either zero or one new vertex to $H_0$. Thus, it is trivial to classify external transitions by their usefulness (0 or 1). Therefore, let us suppose that there are multiple connected components in *H* and denote them by $H_0$ (which contains *r*), $H_1$, $H_2$, $\dots$, see Fig. 2.

Now, let us add the set of external transitions to graph *H*, and let us denote the graph we obtain by *G*. Figure 2 shows an example graph *G* where the internal and external transitions correspond to solid and dotted edges, respectively. Note that the grey vertex between subgraphs $H_0$ and $H_4$ represents a quantum state that is not connected to any internal transitions.

Figure 2 shows various scenarios how to reach the other subgraphs from $H_0$ through external edges:

- to reach subgraph $H_1$ from *r* travelling through at least one external edge is required;
- the same is true for subgraph $H_2$; however, there is an alternative solution to reach both $H_1$ and $H_2$: use the edge between $H_1$ and $H_2$, and either the $H_0$–$H_1$ edge or the $H_0$–$H_2$ edge;
- the path from *r* to subgraph $H_3$ uses at least two external edges, but observe that this path also goes through $H_2$;
- subgraph $H_4$ can be reached through two external edges, where the midpoint vertex is not in *H* (*i.e.*, no internal transition has the midpoint vertex as an endpoint);
- finally, to reach subgraph $H_5$, only one external edge is required, but there are two options to pick this one from.

**Fig. 3:**
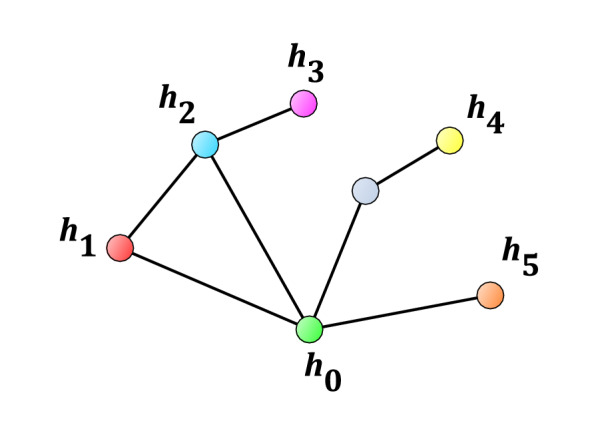
Construction of graph $G'$ from *G*, of Fig. 2, by contracting the subgraphs $H_0$, $H_1$, ..., to single vertices $h_0, h_1, \dots$, respectively, then contracting the parallel edges to single edges

Now, let us contract each $H_0, H_1, \dots$ subgraph to single vertices $h_0, h_1, \dots$. Then, contract parallel edges between vertex pairs to single edges. Let us denote this graph by $G'$. Figure 3 shows the graph obtained after performing this step on the graph of Fig. 2. Note that $G'$ contains only edges that correspond to external transitions. Basically, the CSC method will determine the usefulness of edges or edge sets of $G'$, and the final step is to look up the corresponding external transitions.

Before we continue with the description of the CSC method, let us stop to show a *global* solution. Determining the minimum number of external edges to add to the graph to connect all $H_i$ subgraphs to $H_0$ has a straightforward solution: determine the minimum weight spanning tree [19] of $G'$. After the addition of external transitions to the database, where each edge of the minimum weight spanning tree correspond to one new transition, the result is a connected graph. The problem with the global solution is that the result might be too complex for practical use. Therefore, let us continue the description of the CSC method, which will yield a *local* solution.

In the next step of the CSC method, let us find the shortest paths in $G'$ from $h_0$ to all other vertices [21]. Let us denote the shortest path length in $G'$ from $h_0$ to $h_i$ by $l_i$. For example, in Fig. 3, $l_1=l_2=l_5=1$ and $l_3=l_4=2$.

We need not only the lengths of each of the shortest paths, but we do require all shortest paths (*i.e.*, edge lists that correspond to the paths), as well. Finding all shortest paths between vertex pairs can be done, for example, using a version of the breadth-first-search (BFS) algorithm [21].

Let us store the shortest paths between vertices $h_0$ and $h_i$ in the set $S_i$. The elements of $S_i$ correspond to paths and each path can be represented by its edge set. Thus, $S_i$ is a set of sets, as follows. In the trivial case of $l_i=1$, there is only one set in $S_i$, corresponding to the path whose set has only one edge in it, *e.g.*, $S_i=\{ \{e_1\} \}$. If $l_i>1$, then each path for $S_i$ is represented by the set of its edges, for example (for $l_i=2$): $S_i=\{ \{e_1, e_2\}, \{e_3,e_4\}, \dots \}$.

Finally, let us define a utility factor $u_i$ for each $H_i$, $i>0$, as follows:

$$\begin{aligned} u_i = \frac{|H_i|}{l_i}, \end{aligned}$$

where $|H_i|$ is the number of vertices in $H_i$. In Fig. 2, for example, we see that

$$\displaystyle u_1=\frac{4}{1}=4, \quad u_2=\frac{3}{1}=3, \quad u_3=\frac{2}{2}=1,$$

$$\displaystyle u_4=\frac{4}{2}=2, \quad u_5=\frac{4}{1}=4.$$

### Output

Let us recall that the goal of CSC is to determine a ranking among external transitions (or transition sets), which reflects their usefulness when adding them to the set of internal transitions. According to the CSC method, these suggested transition sets are those which correspond to the $S_i$ sets, each having a utility factor of $u_i$, whereby a higher utility factor means a more useful transition set.

To obtain an output that is ready for practical use, the final step of the algorithm is to find the transitions corresponding to the $S_i$ paths. (For example, $S_5$ contains only one edge, but this edge represents two transitions, see Fig. 2.)

Let us observe how the output corresponding to our example graph in Fig. 2 looks like:

1. The addition of either the $H_0$–$H_1$ transition or one of the two $H_0$–$H_5$ transitions is the most useful. In each case, we would reach four new vertices.
2. Adding the $H_0$–$H_2$ transition is the second most useful expansion of the original graph, yielding three new vertices.
3. The next set of transitions is the two-length path to connect $H_0$ and $H_4$. Here, adding two transitions brings in four new vertices.
4. The least useful transition set is the two extra transitions that connect $H_0$ and $H_3$, offering the two new vertices of $H_3$. Note that by adding these two edges we also connect $H_2$ with its three vertices to $H_0$, making this a more useful expansion than it seems. See the *On the utility factor* section for the elaboration of this phenomenon.

In practice (see the next section), there are utility factors of real examples as high as 91.5.

### Remarks

#### On the utility factor

The CSC algorithm proposed is a bit too strict, at least in the sense that while connecting $H_3$ would also connect $H_2$, the extra vertex contribution by $H_2$ is not reflected in $u_3$. Thus, the above definition of the utility factor can be viewed as a lower bound: some paths (transition sets) might actually be more useful than shown by their utility factor.

By modifying the formula of the utility factor, this phenomenon can also be incorporated into the calculations. However, this yields additional issues: for example, what if one of the shortest paths to $H_a$ goes through $H_b$, while another shortest path to $H_a$ goes through $H_c$? These scenarios could easily make the big picture inconveniently blurry.

Therefore, we advocate another approach to gain insight while keeping the utility factor formula simple. If there are at least three, relatively large $H_i$, $i>0$ subgraphs for which $l_i>1$, then alongside the local solution provided by the CSC algorithm, also calculate the global solution. Then, use both outputs and form the final transition set suggestion by manually selecting transition sets to connect the $H_i$ components to $H_0$.

Note that the balance between the local and global solution is that while the global solution connects all $H_i$ subgraphs using the minimum number of new transitions, the local solution is more resistant to problems that may occur with the external transitions after using the CSC method. For example, let us assume that in Fig. 3, the global solution to connect $h_0$ to $h_2$, then $h_2$ to both $h_1$ and $h_3$ has been selected (we omit in this example the connection of $h_4$ and $h_5$). If it turns out that the selected $h_0$-$h_2$ transition cannot be measured, then this collapses the connection of three components. In contrast, let us take a look at the local solution where we try to connect $h_0$ directly to both $h_1$ and $h_2$, then connect $h_3$ to $h_2$. If problems occur with the measurement of the $h_0$-$h_2$ transition, it does not affect the connection of the component corresponding to $h_1$. Finding the balance between the local and global approaches is left to the user, with a remark that we advocate using primarily the local solution.

#### On not using graph contractions

Another way to think about what is happening during graph construction, but without actually using contractions, is as follows. Let us treat the graph of Fig. 2 as a weighted graph, with solid edges having a weight of 0 and dotted edges having a weight of 1. Then, find the shortest paths from *r* to one vertex from each $H_i$ subgraph. Theoretically, we obtain the same $S_i$ transition sets this way. However, there are two disadvantages of not using graph contractions:

1. The graph algorithms used here, most notably BFS, scale with the number of vertices and edges in the input graph. By contracting potentially large graphs into single vertices, we obtain a huge run-time improvement.
2. Additional care should be exercised in finding all shortest paths when there are edges with zero weight in the graph. In the CSC method, we avoid this problem by finding shortest paths in an unweighted graph.

#### On reducing time and space complexity

The main idea of reducing the running time of the algorithm is to disregard ‘small’ $H_i$, $i>0$ subgraphs when finding the shortest paths. We advocate to disregard the $H_i$, $i>0$ subgraphs for which $|H_i|<8$. This greatly speeds up the algorithm.

One can also introduce a lower bound for the utility factor to use for the output, thus avoiding extremely large output files. We advocate a lower bound around 8 to 10.

#### On finding accurate floating components

A niche use of the CSC method is to find ‘accurate floating components’. This term, introduced here, refers to either a floating component or a subgraph of a floating component, which is composed of accurate transitions.

Typically, spectroscopic databases contain floating components, but these accurate (sub-)graphs are masked by the fact that accuracy has not been checked before. A subgraph containing 10 accurate (*e.g.*, with an uncertainty lower than $10^{-6}$ cm$^{-1}$) transitions could easily hide in a floating component of 15 vertices – however, disregarding accuracy, this floating component does not come up as interesting (*i.e.*, worth connecting to the large main component by adding new transitions).

In order to determine accurate floating components, the connected components of (1) the complete SN and (2) the SN composed of only the accurate transitions, should be compared. However, as determining the connected components is already part of the CSC method, one could also creatively use it for this task, by comparing the components obtained at two uncertainty thresholds: first, the desired accuracy (*e.g.*, $10^{-6}$ cm$^{-1}$), and second, $10^0$ cm$^{-1}$ (whereby all transitions are accurate).

Let us call briefly an accurate floating component *sufficiently large* if it contains at least 5 vertices connected with transitions that have their uncertainty under $5 \times 10^{-6}$ cm$^{-1}$, or if it contains at least 30 vertices connected with transitions that have their uncertainty under $10^{-5}$ cm$^{-1}$. With regard to the next, *Practical examples* section, there are no sufficiently large accurate floating components of H$_2{}^{16}$O, H$_2^{~12}$C$^{16}$O, $^{32}$S$^{16}$O$_2$, and $^{14}$NH$_3$.

## Practical examples

In this section we investigate how the CSC algorithm suggests transitions for re-measurement to improve the accuracy by which we know the absolute energies of quantum states. The use of the CSC method is illustrated on spectroscopic data of four molecules: H$_2{}^{16}$O, $^{32}$S$^{16}$O$_2$, H$_2^{~12}$C$^{16}$O, and $^{14}$NH$_3$. In all cases, the origin of the spectroscopic data is the ReSpecTh spectroscopic information system [5]. In the case of $^{14}$NH$_3$, two extra lines, often referred to as “magic numbers” [15, 16, 22], were added to the ReSpecTh transition set, with an uncertainty value of $1 \times 10^{-6}$ cm$^{-1}$: a transition between the root $(0, 0, 0, 0, 0, 0, 0, 0, s, A1', 1)$ and the state $(0, 0, 0, 0, 0, 0, 1, 1, s, E'', 1)$, and another transition between the root $(0, 0, 0, 0, 0, 0, 0, 0, s, A1', 1)$ and the state $(0, 0, 0, 0, 0, 0, 0, 0, a, A2'', 1)$ [for the labels of the quantum states, here and below, see the original publication(s)].

**Table 1:** Overview of the spectroscopic and graph-theoretical characteristics of the four molecules selected for this study, H$_2^{~16}$O [23], $^{32}$S$^{16}$O$_2$ [24], H$_2^{~12}$C$^{16}$O [25], and $^{14}$NH$_{3}$ [26]; unc. = uncertainty, $|H_i|$ is the number of vertices in the $H_i$ subgraph, $u_i$ is $|H_i|/l_i$, where $l_i$ is the shortest path length from $h_0$ to $h_i$, and $h_i$ is the contraction of $H_i$ to a single vertex

Molecule	Unc. threshold	$|H_0|$	Top suggestions			
	(cm$^{-1}$)		$u_i$	$|H_i|$	$l_i$	Transition pool, if $l_i=1$
H$_2^{~16}$O	$1\times 10^{-6}$	165	No suggestions			
	$5 \times 10^{-6}$	207	43	43	1	405
			39	39	1	330
	$1\times 10^{-5}$	212	45	45	1	424
			43	43	1	360
			29	29	1	267
	$5 \times 10^{-5}$	910	No suggestions			
	$1\times 10^{-4}$	1038	No suggestions			
	$5 \times 10^{-4}$	4274	86	86	1	26
	$1\times 10^{-3}$	4831	88	88	1	27
	$5 \times 10^{-3}$	15 278	14	14	1	2
$^{32}$S$^{16}$O$_2$	$1 \times 10^{-6}$	3	32	32	1	1
			8	16	2	–
	$5 \times 10^{-6}$	139	59	118	2	–
			27	27	1	6
	$1 \times 10^{-5}$	157	71	142	2	–
			29	29	1	11
			28	28	1	36
	$5 \times 10^{-5}$	405	91.5	183	2	–
			56	56	1	89
	$1 \times 10^{-4}$	407	91.5	183	2	–
			91	91	1	168
	$5 \times 10^{-4}$	14216	No suggestions			
	$1 \times 10^{-3}$	14928	No suggestions			
	$5 \times 10^{-3}$	15129	No suggestions			
H$_2^{~12}$C$^{16}$O	$1 \times 10^{-6}$	8	12	12	1	1
			8	8	1	3
	$5 \times 10^{-6}$	37	35.5	71	2	–
			14.5	29	2	–
			14	14	1	43
			14	14	1	35
			13	13	1	30
	$1 \times 10^{-5}$	37	36	72	2	–
			14.5	29	2	–
			14	14	1	43
			14	14	1	35
			13	13	1	30
	$5 \times 10^{-5}$	41	36	72	2	–
			14.5	29	2	–
			14	14	1	43
			14	14	1	35
			13	13	1	30
	$1 \times 10^{-4}$	41	36	72	2	–
			14.5	29	2	–
			14	14	1	43
			14	14	1	35
			13	13	1	30
	$5 \times 10^{-4}$	3699	12	12	1	3
	$1 \times 10^{-3}$	4221	18	18	1	3
	$5 \times 10^{-3}$	4981	No suggestions			
$^{14}$NH$_{3}$	$5 \times 10^{-6}$	151	14	14	1	6
	$1 \times 10^{-5}$	153	38	38	1	293
			30	30	1	299
	$5 \times 10^{-5}$	192	38	38	1	349
			30	30	1	366
			28	28	1	41
	$1 \times 10^{-4}$	512	86	86	1	76
	$5 \times 10^{-4}$	2497	20	20	1	4
	$1 \times 10^{-3}$	3663	32	32	1	1
	$5 \times 10^{-3}$	4649	53	53	1	3

Table 1 provides an overview of the data generated for the H$_2{}^{16}$O [23], $^{32}$S$^{16}$O$_2$ [24], H$_2^{~12}$C$^{16}$O [25], and $^{14}$NH$_{3}$ [26] molecules. Table 1 is structured as follows. The uncertainty threshold is a lower bound: transitions with at least this uncertainty form the set of the external transitions; transitions with a smaller uncertainty than the threshold form the set of the internal transitions. Note that, in practice, if there are both external and internal transitions between an (*u*, *v*) vertex pair, the external transitions can be removed before running the CSC algorithm. Else, external transitions of this type are removed at the contraction of the (*u*, *v*) edge to a single vertex.

Moreover, $|H_0|$ is the number of vertices in $H_0$ (the subgraph containing the root). The right-hand side of the Table 1 shows the top suggestions provided by the CSC method. The meaning of the $u_i$, $H_i$, and $l_i$ values are described in the previous section ($|H_i|$ is the number of vertices in $H_i$). The rightmost column, labeled “transition pool” is a bit less intuitive: if $l_i=1$, that is, when one new transition would connect the $H_i$ subgraph to $H_0$, then it shows the number of unique, inaccurate transitions in the database, from which one should be re-measured with the required improved accuracy. The $l_i>1$ case is much harder to quantify because of the presence of (non-trivial) paths; thus, in this case, these cells are left blank. A note regarding H$_2^{~12}$C$^{16}$O in Table 1: the top suggestions are the same at the three uncertainty thresholds of $1\times 10^{-5}$, $5\times 10^{-5}$, and $1\times 10^{-4}$ cm$^{-1}.$

### The growth of $|H_0|$

The numbers $|H_0|$ in Table 1 are the number of quantum-state energies reachable under the corresponding accuracy. While examining Table 1, one should observe the rapidly expanding $|H_0|$ values as the uncertainty threshold is increased.

In the case of H$_2{}^{16}$O, the two most notable jumps in $|H_0|$ happen at $10^{-5} \text { cm}^{-1} \rightarrow 5\times 10^{-5} \text { cm}^{-1}$, more than quadrupling in size, and at $10^{-3} \text { cm}^{-1} \rightarrow 5\times 10^{-3} \text { cm}^{-1}$, almost quadrupling in size. In the case of $^{32}$S$^{16}$O$_2$, not counting the jump from 3 to 139, the main growth happens at $10^{-4} \text { cm}^{-1} \rightarrow 5 \times 10^{-4} \text { cm}^{-1}$. The main expansions of H$_2^{~12}$C$^{16}$O and $^{14}$NH$_{3}$ also happen at $10^{-4} \text { cm}^{-1} \rightarrow 5\times 10^{-4} \text { cm}^{-1}$. These jumps, of course, reflect the fact that the majority of the high-resolution transitions available in the ReSpecTh database have been measured in absorption using Fourier-transform infrared (FT-IR) spectroscopy.

### Cases of rapid expansion

$|H_i|$ is the number of new quantum states that would become reachable after the addition of new transitions to the original set. The corresponding expansion of the number of accurate energy levels can be expressed by the ratio $\displaystyle \frac{|H_0|+|H_i|}{|H_0|}$.

For example, this ratio is $\displaystyle \frac{207+43}{207} \approx 1.21$ in the most useful suggestion at the uncertainty threshold of $5 \times 10^{-6}$ cm$^{-1}$ for H$_2^{~16}$O, implying a highly meaningful expansion at the cost of adding just one new accurate transition. In contrast, the top suggestion for the same H$_2^{~16}$O case, but at $5 \times 10^{-3}$ cm$^{-1}$, shows a ratio of $\displaystyle \frac{15278+14}{15278} \approx 1$, indicating a marginal expansion that is perhaps not worth pursuing.

Suggestions for the same molecule and at the same uncertainty threshold can also be considered together. As an example, one can use both suggestions in Table 1 for $^{32}$S$^{16}$O$_2$ at the uncertainty threshold of $10^{-4}$ cm$^{-1}$, and add a total of three new transitions with at most this uncertainty to reach $183+91=274$ new vertices. These three transitions would increase $|H_0|$ from 405 to $405+274=679$, which is approximately a 1.68-fold increase.

### The case of disconnected ultraprecise measurements

For $^{14}$NH$_{3}$, it is worth taking a closer look at the suggestion at the extremely low uncertainty threshold of $5 \times 10^{-6}$ cm$^{-1}$. In this case, $|H_0|=151$ and $|H_i|=14$, corresponding to a possible 1.09-fold increase of $|H_0|$, which is already quite noteworthy at this threshold. A detailed investigation of the corresponding graph structure, however, sheds light on more interesting details and consequences.

**Fig. 4:**
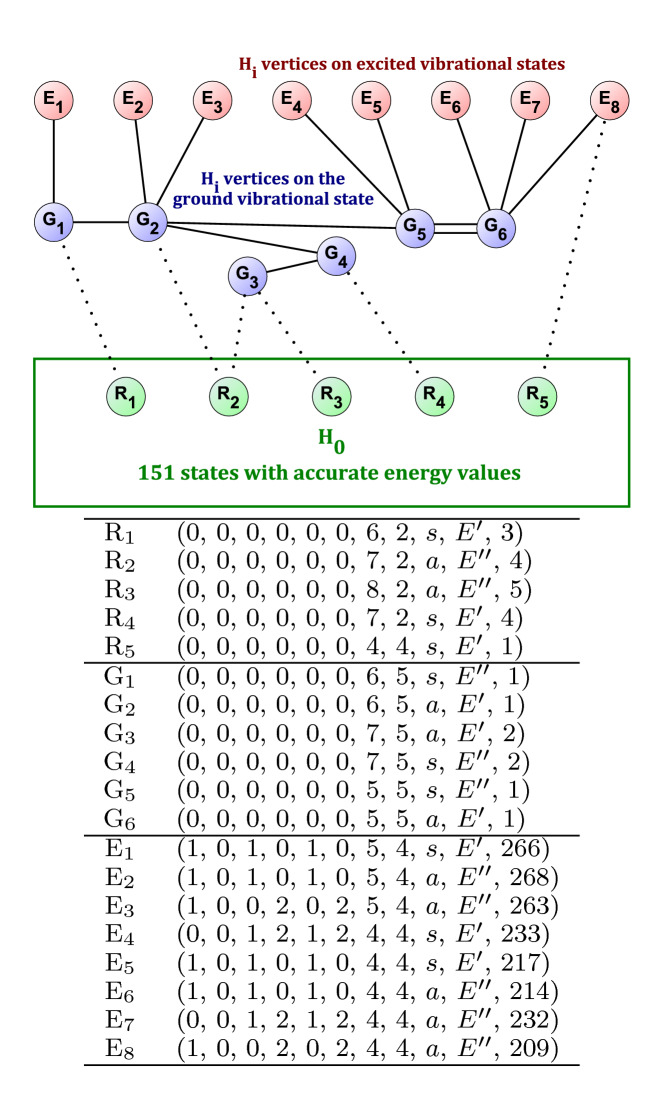
The graph showing the suggestion of Table 1 for $^{14}$NH$_{3}$ at the uncertainty threshold of $5 \times 10^{-6}$ cm$^{-1}$. The R$_i$ vertices are in the same subgraph as the root. The G$_i$ (ground vibrational state) and E$_i$ (excited vibrational state) vertices cannot be reached from the root *via* a path built from accurate transitions

The structure of the graph is shown in Fig. 4, where solid edges represent transitions with uncertainties lower than $5 \times 10^{-6}$ cm$^{-1}$, while dotted edges show the less accurate transitions of the database. The vertices of $H_i$ are colored blue or red, depending on whether they are on the ground vibrational state or not, respectively. The six dotted edges correspond to the number six in the rightmost cell of the related entry of Table 1.

**Table 2:** Format of the files in the Additional file. This segment is from the CSC output corresponding to the $^{32}$SO$_2$ input at the $5\times 10^{-6}$ cm$^{-1}$ uncertainty threshold (see Table 1)

98 1 1558.64590000 0.00148854 0 3 0 2 2 0 0 0 0 1 1 1 17UlBeGrBe.1597	
—	
98 2 1023.54790000 0.00091459 0 3 0 2 2 0 0 1 0 3 3 1 17UlBeGrBe.515	
98 2 1039.36030000 0.00133375 0 3 0 2 2 0 0 1 0 2 1 1 17UlBeGrBe.639	
98 2 1040.73540000 0.00080000 0 3 0 2 2 0 0 1 0 1 1 1 17UlBeGrBe.648	

First column: the index of the path. Second column: the index of the edge in the path. Columns 3+: transitions corresponding to the edge of the path. In practice, this means that one should re-measure the ’17UlBrGrBe.1597’ transition, and one of the three other transitions, to form a new, accurate path, improving the accuracy of 118 new quantum states

It is of interest to note that while all eight ultraprecise transitions between the ground and the excited vibrational states of $^{14}$NH$_{3}$ come from a single source, 16TwHaSe [27], the lower states of these transitions are not connected through ultraprecise measurements to the rest of the pure rotational states. This observation proves the considerable utility of the network approach to spectroscopy, as it reveals an information which otherwise would remain hidden in the observed transitions. In the language of graph theory, the determination of highly accurate ultraprecise absolute energies requires highly accurate paths to the root. Thus, since there is no “solid” path from the root to $H_i$, the ultraprecise measurements of 16TwHaSe [27] do not contribute (yet) towards the goal of determining ultraprecise energies.

Additionally, this $H_i$ subgraph remains disconnected from the root at the $1 \times 10^{-5}$ cm$^{-1}$ and even at the $5 \times 10^{-5}$ cm$^{-1}$ uncertainty thresholds. The vertices of the subgraph can only be reached from the root at the next uncertainty threshold, that is $1 \times 10^{-4}$ cm$^{-1}$. Thus, the energy value of these 14 quantum states inherit an uncertainty of $1 \times 10^{-4}$ cm$^{-1}$, despite participating in transitions with uncertainties less than $5 \times 10^{-6}$ cm$^{-1}$.

Obtaining accurate experimental data for any of the six suggested transitions would connect the 14 states to the root, providing ultraprecise absolute energies for 14 more rovibrational states.

### Cases of single transition suggestions

There are three occurrences in Table 1 where $l_i=1$ and $|S_i|=1$. This means that only one transition with at least the required accuracy should be added, but it should be picked from a set containing just a single transition. (Thus, there is no set of 400+ possible transitions to pick one from, as in the case of H$_2^{~16}$O at $5 \times 10^{-6}$ cm$^{-1}$ and at $1 \times 10^{-5}$ cm$^{-1}$.) Given that two of these three simple suggestions also seem very useful, we opted to include their detailed discussion here as three detailed examples.

The top suggestion for $^{32}$S$^{16}$O$_2$ at $1 \times 10^{-6}$ cm$^{-1}$ designates the transition $(0, 0, 0, 3, 1, 3) \leftarrow (0, 0, 0, 2, 0, 2)$ to add to the database with improved accuracy. This transition has the reference tag ‘78Lovas.519’. After this addition, $H_0$ would grow from 3 vertices to $3+32=35$ vertices, which is an approximately 11.67-fold increase.

The top suggestion for H$_2^{~12}$C$^{16}$O at $1 \times 10^{-6}$ cm$^{-1}$ designates the transition $(0, 0, 0, 0, 0, 0, 1, 1, 1) \leftarrow (0, 0, 0, 0, 0, 0, 0, 0, 0)$ to add to the database with improved accuracy. This transition has the reference tag ‘97CaHaDe.1’. After this addition, $H_0$ would grow from 8 vertices to $8+12=20$ vertices, which is a 2.5-fold increase.

The top suggestion for $^{14}$NH$_{3}$ at $1 \times 10^{-3}$ cm$^{-1}$ designates the transition $(0, 0, 0, 1, 0, 1, 17, 16, s, A_2', 11) \leftarrow (0, 0, 0, 0, 0, 0, 18, 18, a, A_2'', 1)$ to add to the database with the higher accuracy. This transition has the reference tag ‘84UrCuNaPa.952’. However, here $|H_0|=3663$ and $|H_i|=32$, implying that this would be just a marginal expansion of $H_0$.

### The case of large *J* values

Delving deeper into the data about H$_2^{~16}$O (see the Additional file) brings up a new issue that has not been addressed yet: some transitions are easier to measure than others. For example, for the top suggestion at the $5 \times 10^{-6}$ cm$^{-1}$ threshold, with $|H_0|=207$ and $|H_i|=43$, 76 transitions out of the total of 405 transitions lie between quantum states with *J* values of at most 4. In comparison, all transitions corresponding to the top suggestion at $10^{-3}$ cm$^{-1}$ lie between quantum states with *J* values between 26 and 30.

The best way to avoid issues like these is to manually build the set of external transitions, based on the measurement preferences, then run CSC with this set. After this refinement of the input, the output would also consist of transitions feasible for remeasurement.

### The case of identifying critical wavenumber regions

CSC outputs can also be used to find the most useful measurement interval of fixed length *L* (*e.g.*, $L=100$ cm$^{-1}$). A straightforward method to do this is as follows.

Let us run the CSC algorithm, and sort all suggested external transitions in an ascending order based on their respective wavenumbers to obtain the ordering $t_1, t_2, t_3, ...$. Then, let $T_i$ denote the number of $H_i$ components that could be reached using the transitions within the $[t_i, t_i+L]$ interval.

For the highest $T_i$ value obtained, let us denote $t_i=A$, and let the wavenumber of the transition with the highest wavenumber value of the $[A, A+L]$ interval be *B*. Then, the most useful wavenumber interval has to include both wavenumber values *A* and *B* (note that $B-A< L$). If the highest $T_i$ value is not unique but occurs for multiple indices, then we have multiple intervals that are the most useful.

Similarly, wavenumber intervals which do not contribute towards the main goal at all can also be highlighted. For example, let us consider the database of the $^{14}$NH$_{3}$ molecule [26]. At both uncertainty thresholds of $5 \times 10^{-6}$ cm$^{-1}$ and $1 \times 10^{-5}$ cm$^{-1}$, CSC does not suggest transitions above 1815.3719 cm$^{-1}$ but one, that has a wavenumber value of 6576.74634 cm$^{-1}$.

### Supplementary material format

The supplementary material of this article consists of CSC outputs based on ReSpecTh inputs of molecules at various uncertainty thresholds. Table 2 shows the structure of the text files of the Additional files 1–31.

## Conclusions

Improving the accuracy of the absolute energies of (rovibronic) quantum states of molecules is an important task itself. The accuracy of the energy values is based on the accuracy of the measured transition wavenumbers. Improved spectroscopic data are obtained likely through spectroscopic measurement techniques with improved precision, sensitivity, and resolution, facilitating the more accurate determination of the center of the resolved lines. It is important to remember that not all transitions contribute equally to the goal of expanding the set of accurately known energy levels. Therefore, it is an outstanding problem how to optimize the set of lines suggested for re-measurement in order to increase the overall accuracy of the dataset in the most efficient way.

Besides the representation of *all* the available transition wavenumbers of assigned rovibronic lines, spectroscopic networks offer a number of advantages and opportunities to solve challenges of high-resolution spectroscopy. For example, the method of Connecting Spectroscopic Components (CSC) introduced in this paper facilitates the optimal selection of transitions to be remeasured in order to improve the accuracy of the rovibronic energy levels of the underlying dataset.

We have shown that the CSC technique is able to suggest useful sets of transitions to measure when the goal is to improve the accuracy of the absolute energies of a significant number of quantum states. Both the database to improve and the set of possible new transitions are defined by the user. This allows experimental research groups to evaluate CSC suggestions regarding various measurement setups (that correspond to different sets of new transitions), and compare their usefulness towards making energy data more accurate in the selected database.

Several practical, worked-out examples, involving the molecules H$_2{}^{16}$O, $^{32}$S$^{16}$O$_2$, H$_2^{~12}$C$^{16}$O, and $^{14}$NH$_{3}$, prove the usefulness and the advantageous features of the CSC method. The prime application of CSC is the detection of opportunities for the rapid expansion of the set of accurately known energies. For example, in the case of the H$_2{}^{16}$O molecule, there are 206 rovibronic quantum states that are connected to the root *via* a path of transitions that have a wavenumber uncertainty smaller than $5 \times 10^{-6}$ cm$^{-1}$. Here, the addition of one new transition out of 405 possible ones, with an uncertainty lower than $5 \times 10^{-6}$ cm$^{-1}$, would connect an additional 43 quantum states in this way, which is a 1.21-fold expansion.

CSC can also find high-precision transitions that do not contribute towards improving energy-level accuracy as effectively as they could. A set of such ultraprecise transitions is presented for the $^{14}$NH$_{3}$ molecule.

Another application of the CSC method is to highlight wavenumber intervals that are dense or sparse in useful lines to measure in order to improve energy-level accuracy. An example of a large wavenumber interval which does not contain suggested transitions is shown for the $^{14}$NH$_{3}$ molecule: for uncertainties of $5 \times 10^{-6}$ cm$^{-1}$ and $1 \times 10^{-5}$ cm$^{-1}$ the wavenumber range starting at 1815.3718 cm$^{-1}$ is extremely sparse, it contains only one line suggested by CSC.

## Supplementary Information

**Additional file 1.** 14NH3_1.10(− 3).txt: CSC output of the ^14^NH_3_ molecule at the 1∗10^−3^ cm^−1^ uncertainty threshold.

**Additional file 2.** 14NH3_1.10(− 4).txt: CSC output of the ^14^NH_3_ molecule at the 1∗10^−4^ cm^−1^ uncertainty threshold.

**Additional file 3.** 14NH3_1.10(− 5).txt: CSC output of the ^14^NH_3_ molecule at the 1∗10^−5^ cm^−1^ uncertainty threshold.

**Additional file 4.** 14NH3_5.10(− 3).txt: CSC output of the ^14^NH_3_ molecule at the 5∗10^−3^ cm^−1^ uncertainty threshold.

**Additional file 5.** 14NH3_5.10(− 4).txt: CSC output of the ^14^NH_3_ molecule at the 5∗10^−4^ cm^−1^ uncertainty threshold.

**Additional file 6.** 14NH3_5.10(− 5).txt: CSC output of the ^14^NH_3_ molecule at the 5∗10^−5^ cm^−1^ uncertainty threshold.

**Additional file 7.** 14NH3_5.10(− 6).txt: CSC output of the ^14^NH_3_ molecule at the 5∗10^−6^ cm^−1^ uncertainty threshold.

**Additional file 8.** 32SO2_1.10(− 3).txt: CSC output of the ^32^S^16^O_2_ molecule at the 1∗10^−3^ cm^−1^ uncertainty threshold.

**Additional file 9.** 32SO2_1.10(− 4).txt: CSC output of the ^32^S^16^O_2_ molecule at the 1∗10^−4^ cm^−1^ uncertainty threshold.

**Additional file 10.** 32SO2_1.10(− 5).txt: CSC output of the ^32^S^16^O_2_ molecule at the 1∗10^−5^ cm^−1^ uncertainty threshold.

**Additional file 11.** 32SO2_1.10(− 6).txt: CSC output of the ^32^S^16^O_2_ molecule at the 1∗10^−6^ cm^−1^ uncertainty threshold.

**Additional file 12.** 32SO2_5.10(− 3).txt: CSC output of the ^32^S^16^O_2_ molecule at the 5∗10^−3^ cm^−1^ uncertainty threshold.

**Additional file 13.** 32SO2_5.10(− 4).txt: CSC output of the ^32^S^16^O_2_ molecule at the 5∗10^−4^ cm^−1^ uncertainty threshold.

**Additional file 14.** 32SO2_5.10(− 5).txt: CSC output of the ^32^S^16^O_2_ molecule at the 5∗10^−5^ cm^−1^ uncertainty threshold.

**Additional file 15.** 32SO2_5.10(− 6).txt: CSC output of the ^32^S^16^O_2_ molecule at the 5∗10^−6^ cm^−1^ uncertainty threshold.

**Additional file 16.** H212C16O_1.10(− 3).txt: CSC output of the H_2_^12^C^16^O molecule at the 1∗10^−3^ cm^−1^ uncertainty threshold.

**Additional file 17.** H212C16O_1.10(− 4).txt: CSC output of the H_2_^12^C^16^O molecule at the 1∗10^−4^ cm^−1^ uncertainty threshold.

**Additional file 18.** H212C16O_1.10(− 5).txt: CSC output of the H_2_^12^C^16^O molecule at the 1∗10^−5^ cm^−1^ uncertainty threshold.

**Additional file 19.** H212C16O_1.10(− 6).txt: CSC output of the H_2_^12^C^16^O molecule at the 1∗10^−6^ cm^−1^ uncertainty threshold.

**Additional file 20.** H212C16O_5.10(− 3).txt: CSC output of the H_2_^12^C^16^O molecule at the 5∗10^−3^ cm^−1^ uncertainty threshold.

**Additional file 21.** H212C16O_5.10(− 4).txt: CSC output of the H_2_^12^C^16^O molecule at the 5∗10^−4^ cm^−1^ uncertainty threshold.

**Additional file 22.** H212C16O_5.10(− 5).txt: CSC output of the H_2_^12^C^16^O molecule at the 5∗10^−5^ cm^−1^ uncertainty threshold.

**Additional file 23.** H212C16O_5.10(− 6).txt: CSC output of the H_2_^12^C^16^O molecule at the 5∗10^−6^ cm^−1^ uncertainty threshold.

**Additional file 24.** H216O_1.10(− 3).txt: CSC output of the H_2_^16^O molecule at the 1∗10^−3^ cm^−1^ uncertainty threshold.

**Additional file 25.** H216O_1.10(− 4).txt: CSC output of the H_2_^16^O molecule at the 1∗10^−4^ cm^−1^ uncertainty threshold.

**Additional file 26.** H216O_1.10(− 5).txt: CSC output of the H_2_^16^O molecule at the 1∗10^−5^ cm^−1^ uncertainty threshold.

**Additional file 27.** H216O_1.10(− 6).txt: CSC output of the H_2_^16^O molecule at the 1∗10^−6^ cm^−1^ uncertainty threshold.

**Additional file 28.** H216O_5.10(− 3).txt: CSC output of the H_2_^16^O molecule at the 5∗10^−3^ cm^−1^ uncertainty threshold.

**Additional file 29.** H216O_5.10(− 4).txt: CSC output of the H_2_^16^O molecule at the 5∗10^−4^ cm^−1^ uncertainty threshold.

**Additional file 30.** H216O_5.10(− 5).txt: CSC output of the H_2_^16^O molecule at the 5∗10^−5^ cm^−1^ uncertainty threshold.

**Additional file 31.** H216O_5.10(− 6).txt: CSC output of the H_2_^16^O molecule at the 5∗10^−6^ cm^−1^ uncertainty threshold.

## Data Availability

The datasets generated during the current study are available in the Additional file. The corresponding input data are available in the ReSpecTh website http://www.respecth.hu/.
